# Dynamic Changes in the Nigrostriatal Pathway in the MPTP Mouse Model of Parkinson's Disease

**DOI:** 10.1155/2017/9349487

**Published:** 2017-07-31

**Authors:** Dongping Huang, Jing Xu, Jinghui Wang, Jiabin Tong, Xiaochen Bai, Heng Li, Zishan Wang, Yulu Huang, Yufei Wu, Mei Yu, Fang Huang

**Affiliations:** ^1^The State Key Laboratory of Medical Neurobiology, The Institutes of Brain Science and the Collaborative Innovation Center for Brain Science, Shanghai Medical College, Fudan University, 138 Yixueyuan Road, Shanghai 200032, China; ^2^School of Life Science and Technology, Tongji University, 1239 Siping Road, Shanghai 200092, China; ^3^School of Basic Medical Sciences, Fudan University, 138 Yixueyuan Road, Shanghai 200032, China

## Abstract

The characteristic brain pathology and motor and nonmotor symptoms of Parkinson's disease (PD) are well established. However, the details regarding the causes of the disease and its course are much less clear. Animal models have significantly enriched our current understanding of the progression of this disease. Among various neurotoxin-based models of PD, 1-methyl-4-phenyl-1,2,3,6-tetrahydropyridine (MPTP) mouse model is the most commonly studied model. Here, we provide an overview of the dynamic changes in the nigrostriatal pathway in the MPTP mouse model of PD. Pathophysiological events, such as reductions in the striatal dopamine (DA) concentrations and levels of the tyrosine hydroxylase (TH) protein, depletion of TH-positive nerve fibers, a decrease in the number of TH-positive neurons in the substantia nigra pars compacta (SNpc), and glial activation, are addressed. This article will assist with the development of interventions or therapeutic strategies for PD.

## 1. Introduction

Parkinson's disease (PD) is the second most common neurodegenerative disorder, which is prevalent among the elderly [1, 2]. An anatomical circuit, the nigrostriatal pathway, is required for the fine tuning of basal ganglion function. This pathway is preferentially damaged in patients with PD and subsequently contributes to the clinical motor abnormalities, including tremor, muscle stiffness, a paucity of voluntary movements, and postural instability [3]. The main neuropathological features of PD are the loss of dopaminergic neurons in the substantia nigra pars compacta (SNpc) and their projections into the caudate nucleus, as well as the cytoplasmic accumulation of proteinaceous aggregates, Lewy bodies (LBs), which were named after the neurologist Frederic Lewy [4]. During a 200-year period, the characteristic brain pathology and motor and nonmotor symptoms of PD have been well described. However, the etiology and pathogenesis of PD remain unclear [5, 6]. Based on abundant studies of postmortem tissue and various genetic or neurotoxic animal models, the death of dopaminergic neurons has been linked to mitochondrial dysfunction, oxidative stress, neuroinflammation, and insufficient protein degradation [7]. Since the first synthesis of MPTP in the 1970s, MPTP-based animal models have greatly improved our understanding of the cause and course of the disease [8, 9]. Here, we provide a brief review of the dynamic changes observed in the nigrostriatal pathway in the MPTP mouse model of PD.

## 2. Microglia, Astrocytes, and Neurons in the Nigrostriatal Pathway

Microglia and astrocytes are categorized as glial cell populations. In contrast to other glial cells and neurons, which are of neuroectodermal origin, microglia are derived from primitive hematopoiesis in the fetal yolk sac and migrate into the brain during early fetal development [10, 11]. Using ablation approaches in the adult brains, microglia have been shown to possess a repopulation capacity; approximately 1 week after depletion, microglia are restored to their normal density [12–14]. Microglia constitute 5–20% of the total glial cell population in the rodent brains. The basal ganglia and substantia nigra (SN) are among the most densely populated areas [15]. Acting as primary cells that respond to pathogen infection and injury, microglial cells play the roles of resident macrophages in the central nervous system (CNS) [10, 16, 17]. By adopting an “amoeboid” phenotype, activated microglia produce many proinflammatory mediators, including cytokines, chemokines, reactive oxygen species (ROS), and nitric oxide (NO) [10]. The presence of reactive microglia in the SNpc of postmortem human brain tissue was first revealed as early as in 1988, suggesting the involvement of neuroinflammation in the pathogenesis of PD [18, 19].

Astrocytes represent the most abundant glial cell type in the adult brain [20]. The most important functions of astrocytes include the maintenance of water and ion homeostasis, participation in the tripartite synapse, and the formation of the blood brain barrier (BBB) [21]. Proteins that are generally used as astrocyte markers are the intermediate filament protein, glial fibrillary acidic protein (GFAP), the glutamate transporter, GLAST, and the aldehyde dehydrogenase 1 family member L1 (Aldh1L1) [22, 23]. Like microglia, astrocytes respond to inflammatory stimuli such as LPS, IL-1*β*, and TNF-*α* by producing an array of pro- and anti-inflammatory mediators, antioxidants, and neurotrophic factors. Reactive astrogliosis, which is characterized by increased expression levels of GFAP and hypertrophy of the cell body and its extensions, has been observed in affected brain regions of patients with PD [24, 25], indicating the involvement of astrocytes in the immune processes in PD [19].

Under physiological conditions, the crosstalk among neurons, astrocytes, and microglial cells are essential for the homeostasis of the nigrostriatal axis. Astrocytes promote the survival and maintenance of dopaminergic neurons by secreting various neurotrophic factors in the SN. Microglial cells survey the cerebral microenvironment and engulf cell debris. Therefore, both astrocytes and microglia are required for neuronal protection. However, astrocytes and microglial cells are not sufficient for the protection of dopaminergic neurons. Microglia and astrocytes are the main components of the innate immune system in the CNS [26, 27]. When the homeostasis of neurons, astrocytes, and microglial cells is disrupted with the administration of dopaminergic neurotoxins, such as MPTP and 6-hydroxydopamine (6-OHDA), neuroinflammatory activities mediated by activated microglia and reactive astrocytes promote neurodegeneration. Glial activation is indexed as an increase in the number and size of glial cells, as well as the induction of morphological changes.

The effects of exaggerated inflammatory responses on microglia are mainly considered deleterious [28]. Inhibition or attenuation of microglial activation is related to dopaminergic neuroprotection [29–32], with the exception of the report on* IL-6* knockout mice [33]. However, the effects of reactive astrocytes are still controversial. Reactive astrocytes have been implicated in inducing oxidative stress and inflammation, therefore triggering the degeneration of dopaminergic neurons [27]. In contrast, astrocyte activation may suppress neuroinflammation and improve the resistance of dopaminergic neurons in animal models of PD. Many of the results have been revealed using gene targeting strategies in animals in combination with the neurotoxin-based PD models. Genes and their products per se are not necessarily beneficial or detrimental. In glial cells, the expressed proteome coordinately implements glial functions. The ablation of certain genes alters responses to glial activation. For example, a glial deficiency of dopamine D1 receptor or an astrocytic deficiency of dopamine D2 receptor exacerbates dopaminergic neuronal loss in MPTP-challenged mice [34, 35], whereas the activation of glial cells lacking major histocompatibility complex II (MHC II) or Lipocalin-2 (LCN2) is inhibited in mice, and the dopaminergic system is subsequently protected following the MPTP treatment [31, 32].

Astrocytes are activated by damaged dopaminergic neurons [32, 36] or activated microglial cells [28, 31, 37]. Moreover, reactive astrocytes also secrete soluble factors that stimulate microglial activation [10, 27]. Interestingly, the status of astrocyte activation is correlated with dopaminergic neurodegeneration in MPTP mouse models: stronger astrocyte activation is associated with more severe damage to the nigrostriatal pathway [35, 36]; attenuated astrocyte activation is correlated with less dopaminergic neuronal loss [32]; and a lack of astrocyte activation is accompanied by nondopaminergic neurodegeneration [31].

## 3. The MPTP Mouse Model of Parkinson's Disease

Neurotoxins, such as MPTP, 6-OHDA, rotenone, paraquat, paraquat combined with manganese ethylenebisdithiocarbamate (Maneb), reserpine, and lipopolysaccharide (LPS), induce dopaminergic neurodegeneration in animals [7, 38]. Neurotoxic effects of MPTP on humans, monkeys, rodents, zebrafish, and* C. elegans* have been observed. For decades, the MPTP mouse model has been the most commonly used model for elucidating damage to the nigrostriatal pathway in PD.

MPTP is systemically administrated to mice. Due to its lipophilic property, it easily crosses the BBB. Inside the brain, MPTP is converted to the intermediate 1-methyl-4-phenyl-2,3-dihydropyridinium species (MPDP^+^) by glial monoamine oxidase-B (MAO-B) [39, 40]. MPDP^+^ is subsequently oxidized to the toxic metabolite 1-methyl-4-phenylpyridinium (MPP^+^) [41]. MPP^+^ is released from the astrocytes through the organic cation transporter 3 into the extracellular space, where it is taken up by the dopaminergic neurons and terminals via the plasma membrane dopamine transporter [7, 38, 42, 43]. Once MPP^+^ accumulates in dopaminergic neurons, it induces neurotoxicity primarily by inhibiting complex I of the mitochondrial electron transport chain [44], resulting in ATP depletion and oxidative stress mediated by superoxide and NO, followed by neuronal death [3].

The inbred strain C57BL/6 with high MAO-B activity is sensitive to MPTP toxicity. Following exposure to MPTP, the striatal MPP^+^ content reaches the highest value within 2 h and the metabolite is cleared from the brain within 12 h [29, 45–48]. In 1995, Jackson-Lewis and Przedborski developed the acute regimen [49], which is widely adopted in the study of PD. In this case, dynamic changes in the nigrostriatal pathway are examined in the acute MPTP mouse model (four injections of MPTP-HCl at a dose of 18–20 mg/kg with 2 h intervals between injections, 1 mg of MPTP-HCl equal to 0.826 mg of free base MPTP). C57BL/6 mice are euthanized at 90 min to 12 h, 1 day to 9 days, and 42 days to 90 days after the last MPTP administration to analyze immediate, early, and late effects, respectively.

## 4. Immediate Effects of MPTP on the Nigrostriatal Pathway

**Figure 1:**
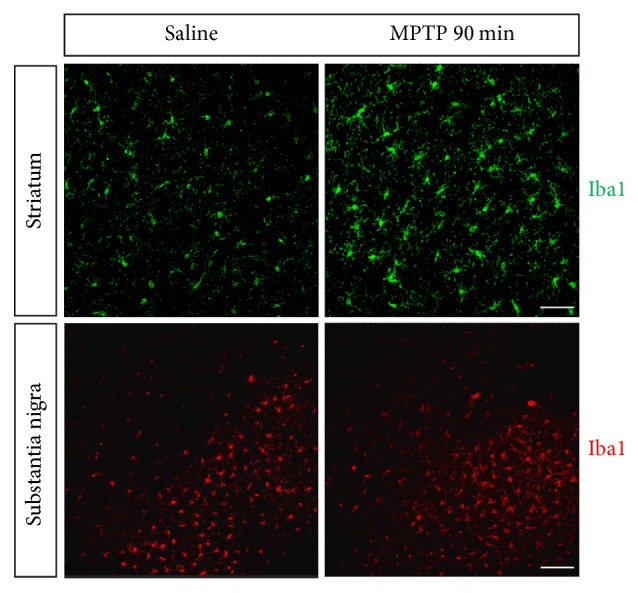
*Microglial cells in the nigrostriatal pathway at 90 min after acute MPTP treatment.* Immunofluorescence staining for Iba1 (green) in the striatum (scale bar: 0.05 mm) and immunofluorescence staining for Iba1 (red) in the SN (scale bar: 0.1 mm) were shown. This figure is adapted from Liu et al.,* scientific reports* 5:15720 [50].

Following an acute MPTP treatment, the striatal concentrations of DA and its metabolite DOPAC are dramatically reduced to approximately 10% of the control level at 90 min after injection. No changes in the striatal HVA, 5-HT, or 5-HIAA levels are observed. At this time point, 67% of the TH-positive (TH^+^) nerve fibers remained intact in the striatum. Thus, the inhibition of the enzymatic activity of TH by MPP^+^ is more dramatic than the degeneration of TH^+^ terminals. Using a stereological counting method, MPTP was shown to cause a slight decrease in the number of TH^+^ neurons in the SNpc (87% of the control). Levels of the TH protein in the SN were comparable between the control and MPTP-injured mice [50]. These findings are consistent with the well-known concept that the striatal TH^+^ fibers have a different sensitivity to the toxin MPTP compared to TH^+^ soma in the SNpc [51–54]. Microglial activation, which is characterized by an increase in the number of microglia and changes in morphology, was detected in the striatum at 90 min after the last MPTP injection [50] (Figure 1). Microglial activation in the striatum and the SN was confirmed 12 h after the acute MPTP treatment in many other studies [55, 56]. Collectively, in the acute MPTP model, systemic injections of MPTP result in a rapid onset of neuroinflammatory responses in the striatum and the SN, and microglial activation and proliferation precede the death of dopaminergic neurons [50, 57].

## 5. Early Effects of MPTP on the Nigrostriatal Pathway

Reductions in striatal levels of the TH protein and the depletion of TH^+^ nerve fibers are observed 1 day after MPTP intoxication. According to studies performing immunohistochemical staining of the midbrain and stereological counting, the numbers of TH^+^ neurons and Nissl-positive neurons are significantly decreased in the MPTP-lesioned SNpc. Notably, TH protein expression is not detected in the Fluoro-Jade B-positive degenerating neurons [50, 58]. At this time point, MPTP induces widespread microglial activation in the nigrostriatal pathway, as indicated by an increase in the number and size of Iba1^+^ microglial cells and morphological changes, such as hypertrophy [50, 59]. At 7 days after injection, a remarkable number of microglial cells were still activated in the SN of young [35, 57] and old mice [60]. One day after MPTP treatment, the striatal levels of the GFAP protein did not change, suggesting the astrocytes might still be at the resting state [58]. In another study, Muramatsu et al. observed a marked increase in the number of GFAP-positive astrocytes (exhibiting a ramified form with many fine processes) in both the striatum and the SN at 3 and 7 days after MPTP treatment [61]. Eight days after MPTP administration, the striatal levels of the GFAP protein were significantly increased [58]. Compared to the responses of microglia and neurons in the acute MPTP model, astrocytes manifest a delayed reaction, which is also involved in mediating the neuroinflammation [57].

**Figure 2:**
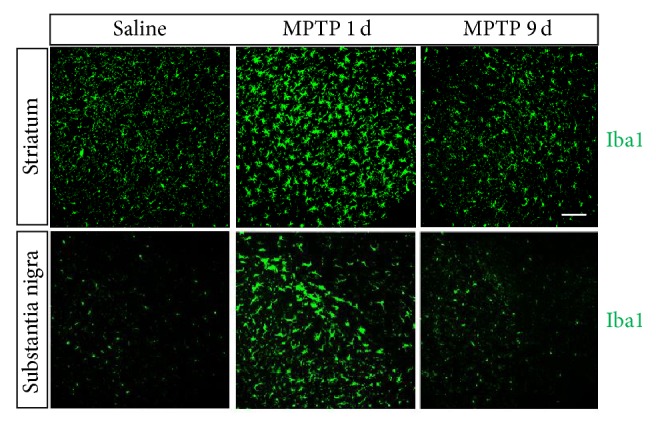
*Microglial cells in the nigrostriatal pathway at 1 and 9 days after acute MPTP treatment.* Immunofluorescence staining for Iba1 (green) in the striatum and in the SN was shown. Scale bar: 0.1 mm. This figure is adapted from Liu et al., scientific reports 5:15720 [50].

At 9 days after the MPTP challenge, the striatal DA concentration reached 17% of the control, which was elevated compared with the value observed at 90 min [50]. The reductions in striatal levels of the TH protein, the depletion of TH^+^ nerve fibers, and the decrease in the number of TH^+^ neurons in the SNpc were maintained. At this time point, the activation of microglia in the nigrostriatal pathway autonomously decreased to a level similar to the resting state [50] (Figure 2).

## 6. Late Effects of MPTP on the Nigrostriatal Pathway

The entire nigrostriatal pathway undergoes a process of recovery 2-3 weeks after the acute MPTP treatment. At 42 days after the last MPTP injection, progressive striatal dopaminergic reinnervation was confirmed by DAT immunoreactivity and [^3^H] dopamine uptake [55]. The striatal levels of the TH protein, the intensity of TH^+^ nerve fibers, and the number of TH^+^ neurons in the SNpc were still significantly reduced (unpublished data). These phenomena are maintained for 90 days after MPTP intoxication. Activated astrocytes are detected in the nigrostriatal pathway at 42 days (unpublished data), 65 days [62], and 90 days after injection (unpublished data).

## 7. Dynamic Changes in the Nigrostriatal Pathway in Subacute, Subchronic, and Chronic MPTP Mouse Models

In mice, acute MPTP treatment primarily damages the nigrostriatal dopaminergic pathway [3]. However, dopaminergic neurons die quickly and little progression in the loss of nigrostriatal DA is observed [46]. According to the study question, different MPTP regimens are applied in different studies [63]. The time course of the deleterious events and the magnitude of the lesion depend on the regimen of administration [52, 64]. Other commonly used MPTP regimens include the following:

1. Presymptomatic PD model: an acute single MPTP application at low dose (1 × 10–20 mg/kg)
2. Subacute PD model: repetitive subacute MPTP applications at intermediate doses (4 × 15–25 mg/kg at 6 or 12 h intervals within two days)
3. Subchronic PD model: daily MPTP injections at doses of approximately 20–30 mg/kg for up to 4-5 consecutive days
4. Progressive chronic PD model: daily injections of low doses (4 mg/kg) of MPTP over 20 days.

In the subacute PD regimen, adult male C57BL/6 mice were challenged with MPTP (24 mg/kg, every 12 h for 2 d). Mice were euthanized at different time points after the last dose. TH protein expression in both the striatum and the SN is noticeably decreased. According to the statistical analysis, the levels of TH protein in the striatum at 18, 36, and 72 h after injection were 38%, 45%, and 31% of the saline group, respectively, and the levels in the SN were 60%, 38%, and 67% of the control, respectively [65]. The dose of MPTP administered to 12-month-old mice was reduced to 15 mg/kg. Four weeks after the MPTP treatment, the level of striatal TH protein decreased by 59% compared with saline-treated mice, whereas the striatal DA concentration after the MPTP treatment decreased by 79%. Approximately 63% of dopaminergic neurons in the SN were lost following the MPTP treatment. The striatal GFAP level increased by 3.7-fold at 3 days after MPTP administration, and this level was maintained for 4 weeks in old mice [36].

The different responses observed in young and old mice have been repeatedly measured, but with some variation. The dosages of MPTP and the age are likely the main determining factors. Young (9–12 weeks old) and old (11–13 months old) mice were intraperitoneally injected with MPTP twice a day at 12 h intervals for 2 days. The dosage of MPTP was 20 mg/kg for young mice and 15 mg/kg for old animals. One or 3 days after the last MPTP injection, MPTP elicited an approximately 90% (1 day) to 55% (3 days) decrease in the striatal levels of the TH protein in young mice. In old mice, MPTP induced a nearly 70% (1 day or 3 days) decrease. On the 8th day, MPTP elicited a 57% decrease in the striatal levels of the TH protein. Approximately 80% and 70% losses of TH^+^ fibers were observed in young mice 1 day or 3 days after MPTP intoxication. Meanwhile, MPTP caused an approximately 66% (1 day) to 58% (3 days) decrease in the density of TH^+^ fibers in old mice. In the SN, MPTP elicited approximately 41% (1 day) to 38% (3 days) and 50% (1 day) to 53% (3 days) loss of TH^+^ neurons in young and old mice, respectively. Based on electron microscopic analyses, MPTP induced a significant increase in the number of structurally altered mitochondria in the SNpc neurons of young mice at 3 days after injection; these alterations comprised numerous vacuoles and fragmented cristae. According to the quantitative analysis, the SNpc neurons exhibited a significantly higher percentage of damaged mitochondria (51.3%) [54]. In a study with cHS4I-hIL-1*β*P-Luc transgenic mice, in which the expression of luciferase reporter gene is controlled by the human* IL-1β* gene promoter [66], both old male and female mice were monitored following subacute MPTP intoxication. MPTP induced elevated expression of the IL-1*β* transcript in the cortex, striatum, and ventral midbrain at 2 h after treatment. Luciferase expression was significantly elevated at 2, 8, 32, and 49 h in MPTP-treated male mice, the inducible signals peaked at 8 h. The old female mice showed a marked increase in luciferase expression at 4 and 26 h after MPTP administration. At 96 h after the last MPTP injection, striatal levels of the GFAP protein were robustly increased. As expected, MPTP elicited less dopaminergic toxicity in old female than in male mice [67].

In the subchronic PD regimen (30 mg/kg/day for 5 consecutive days), MPTP induced the depletion of more than 90% of the TH protein in the striatum and reduced the number of TH^+^ neurons in the SNpc by 30% at 24 h after injection. Notably, MPTP administration significantly increased the expression of *α*-synuclein in the striatum ([68] and unpublished data). The observation of reactive gliosis is also compelling in this model. A significantly higher number of Iba 1-positive cells were observed in the SNpc at 1 day [31] or 2 days [30], after MPTP treatment. However, at 2 days after injection, astrogliosis is not consistently detected [30, 31]. Three weeks after the injection, the striatal DA level was reduced to 17% of the control level by the MPTP treatment [31], whereas 42% of the TH^+^ neurons in the SNpc and 14% of TH^+^ fibers (by density assay) in the striatum were preserved and the astroglial cell count increased [30].

In summary, the specific and reproducible neurotoxic effect on the nigrostriatal system are a strength of MPTP induced PD models. However, MPTP mouse models exhibit an apparent lack of Lewy body-like inclusions bodies in the midbrain [46, 69]. Here, the communication between the CNS and immune system, which also contributes to the progression of PD, should be mentioned. Peripheral immune cells play an important role during the course of neuroinflammation in the mouse MPTP models, with reports of CD4^+^ and CD8^+^ T-lymphocytes infiltrating the SN [70].

## 8. Conclusions

**Figure 3:**
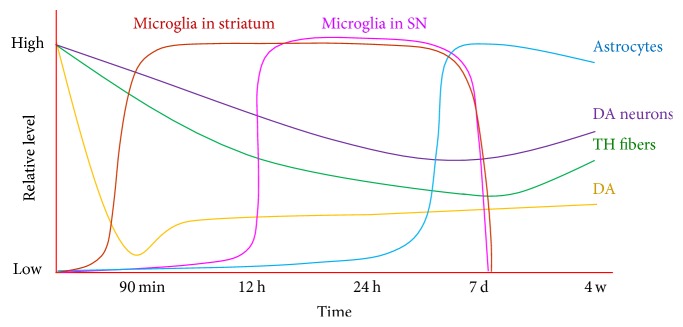
Dynamic changes in the nigrostriatal pathway in the acute MPTP mouse model of Parkinson's disease.

The nigrostriatal pathway plays an important role in regulating the functions of the basal ganglion. Under physiological conditions, neurons, astrocytes, and microglia support each other and maintain a triple “win-win” relationship. When exposed to environmental or endogenous toxins, which may be combined with genetic susceptibility, this circuit is damaged, causing motor symptoms in patients with PD. The strengths and limitations of MPTP mouse models of PD are both remarkable. Microglial activation precedes the degeneration of dopaminergic neurons and astrocyte activation in the nigrostriatal pathway of the acute MPTP mouse model. The dynamic changes observed in the nigrostriatal pathway are summarized in Figure 3. A better understanding of the time course of pathophysiological events will benefit studies developing interventions or therapeutic strategies for PD.
